# Magnetic resonance microscopy of samples with translational symmetry with FOVs smaller than sample size

**DOI:** 10.1038/s41598-020-80652-z

**Published:** 2021-01-12

**Authors:** Igor Serša

**Affiliations:** Department of Condensed Matter Physics, Jožef Stefan Institute, Jamova 39, 1000 Ljubljana, Slovenia

**Keywords:** Techniques and instrumentation, Imaging techniques, Microscopy, NMR spectroscopy

## Abstract

In MRI, usually the Field of View (FOV) has to cover the entire object. If this condition is not fulfilled, an infolding image artifact is observed, which suppresses visualization. In this study it is shown that for samples with translational symmetry, i.e., those consisting of identical objects in periodic unit cells, the FOV can be reduced to match the unit cell which enables imaging of an average object, of which the signal is originated from all unit cells of the sample, with no punishment by a loss in signal-to-noise ratio (SNR). This theoretical prediction was confirmed by experiments on a test sample with a 7 × 7 mm^2^ unit cell arranged in a 3 × 3 matrix which was scanned by the spin-echo and by single point imaging methods. Effects of experimental imperfections in size and orientation mismatch between FOV and unit cell were studied as well. Finally, this method was demonstrated on a 3D periodic sample of tablets, which yielded well-resolved images of moisture distribution in an average tablet, while single tablet imaging provided no results. The method can be applied for SNR increase in imaging of any objects with inherently low signals provided they can be arranged in a periodic structure.

## Introduction

Magnetic resonance imaging (MRI) is one of the most important radiological modality nowadays due to its exceptional contrast among different soft tissues^1,2^, while magnetic resonance microscopy (MRM) is a technique used in MRI for optimized scanning of smaller samples at a higher spatial resolution^3^. Typical applications of MRM are in medicine and biology, e.g., imaging of tissue samples and smaller animals^4–6^, as well as in material science^7^ where applications range from porous materials^8^, energy storage^9^, pharmaceutical research^10,11^ to food science^12^ and wood science^13^. Resolution increase of MRM over standard clinical MRI is for up to two orders of magnitude; i.e., from a millimeter to approximately ten micrometers. In few studies, a micrometer resolution was already reported^14^. First successful MRM of a single animal cell was done in 1986 on an egg cell of African toad^15^. With the application of microcoils, the MRM of other biological cells e.g. mammalian neurons also became possible^16–18^. However, the increase in MRM resolution over the years^19^, is still not comparable to that of optical microscopy. Therefore, routine studies of tissues on a cellular level, investigations of colloid suspensions, liquid crystals and other similar structures on a micrometer scale are still very challenging if not impossible.

In the past, several attempts have been made to improve the resolution of MRM by developing new methods. One of these is magnetic resonance force microscopy (MRFM)^20^, which combines principles of MRI and atomic force microscopy (AFM)^21^. In MRMF, instead of inductive detection, a cantilever tipped with a ferromagnetic material is used to sense a periodic magnetic force of nuclear spins precessing in a magnetic field gradient of the tip. With this approach, detection sensitivity is improved by a factor of 10^10^ over MRI used for medical purpose, making it feasible for the detection of a single spin^22^. However, with MRFM signal detection is restricted only to the sample surface. More straightforward approaches, where the signal is acquired from the entire sample, includes the use of: radiofrequency (RF) microcoils^23,24^, cryoprobes^25,26^, stronger magnetic field magnets^27,28^, and hyperpolarization^29^. The use of microcoils relies on the principle of reciprocity^30^ according to which smaller (micro) coils have a better sensitivity than the large ones. With cryoprobes, the improvement in signal-to-noise ratio (SNR) is due to the noise reduction, which is possible because of reduction in the coil temperature and therefore its thermal noise. The most expensive approach is the use of higher field magnets. In a higher field magnet, the increase in the thermal equilibrium magnetization $M_{0}$ and Larmor precession frequency $\omega_{0}$ is proportional to the increase of magnetic field $B_{0}$. Therefore, the increase in MR signal is proportional to $B_{0}^{2}$. Unfortunately, SNR does not follow this trend because noise also increases with $B_{0}$ due to an increase in coil/sample resistance, so that ${\text{SNR}} \propto B_{0}^{7/4}$ with smaller samples and ${\text{SNR}} \propto B_{0}$ with larger samples^31,32^. Additional problems in higher-field NMR/MRI are also in an increased susceptibility effects and reduced *T*_2_ relaxation times at higher fields. Hyperpolarization results in polarization of nuclei much higher than in thermal equilibrium; however, it can be applied only to specific types of samples and is technically demanding. Hyperpolarization can be performed using various techniques which include: dynamic nuclear polarization^33–35^, spin-exchange optical pumping^36–38^, and parahydrogen induced polarization^39,40^.

The main reason why spatial resolution in MRM cannot be increased much beyond its current limit is inherently associated with the signal-to-noise ratio (SNR) and its scaling properties; i.e., simultaneous reduction in the sample size, field of view (FOV) and RF probe size by the same factor *f,* while other imaging parameters (imaging sequence, matrix, time constants …) remain unchanged. Such reduction would result in the increased resolution by the factor *f*, but decrease in SNR by the factor $f^{2}$. It can be shown that the SNR can be kept constant with the scaling if the static magnetic field is increased by the factor $f^{8/7}$ or signal averaging is increased by the factor $f^{4}$^30,31^. The first approach is expensive while the second one is very time demanding. The increased resolution in MRM is not the only case when signal from a voxel can become too low to enable imaging. The same problem is also encountered when sample signal is low due to the low proton density or very short *T*_2_ relaxation times, e.g., in the imaging of pharmaceutical tablets^11,41^, and nuclei other than the protons. In all these cases, a more efficient method of signal amplification is highly desirable.

Few attempts in the past were undertaken to utilize the advantage of the samples containing arrangement of similar substructures. It was demonstrated that a significant SNR improvement in the images of such samples can be obtained by employing a prior information about the arrangement^42^. The concept of NMR diffraction in solids was proved using an artificial periodic structure^43^. In this study, another application of similar samples arrangement is therefore proposed, namely for the MRI signal amplification. The method implies the use of samples with translational symmetry that are composed of periodically repeating identical objects, in each unit cell of the sample. In the study, it is shown that, if such a sample is MR scanned at a reduced field of view that matches the unit cell of sample both in size and orientation, then the signal is amplified by the number of the objects in the sample (provided that RF probe sensitivity is ideally homogeneous), while the noise remains at the level of conventional imaging of a single object. This effect is first analyzed theoretically then verified by experiments on a test sample and finally it is demonstrated on an artificially made 3D periodic sample of pharmaceutical tablets by imaging moisture content distribution in an average tablet.

## Theory

**Figure 1:**
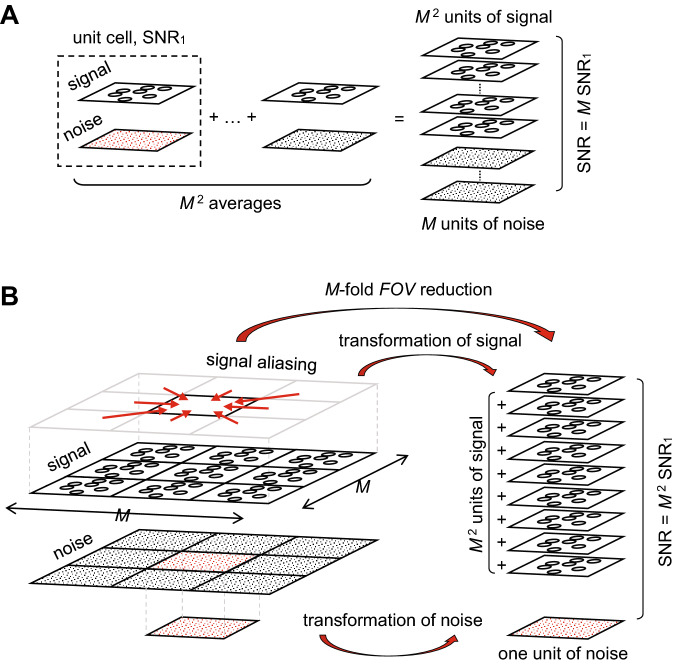
Standard signal averaging vs. constructive signal aliasing. Schematic presentation of the mechanism of SNR increase (**A**) in standard signal averaging and (**B**) in constructive signal aliasing. When signals of $M^{2}$ images are averaged the signal increases by a factor of $M^{2}$, however, noise also increases by a factor of *M*, so that the SNR increase is *M*-fold. In the constructive signal aliasing approach, imaging of a periodic sample with unit cells arranged in a *M* × *M* matrix results in the signal increase by a factor of $M^{2}$, while there is no change in image noise, so that the SNR increase is $M^{2}$-fold.

The simplest way to increase SNR in MRI is by signal averaging. In signal averaging, image signal increases by the factor equal to the number of signal averages, while image noise, as a stochastic event, increases by the factor equal to the square root of the number of signal averages. Therefore, the SNR also increases by the factor equal to the square root of the number of signal averages; in case of *M*^2^ signal averages the SNR increase is *M*-fold (Fig. 1A).

In conventional MRI, a sample is scanned with the field of view (FOV) such that the entire sample or at least in the phase-encoding direction lies within the FOV. If the sample is larger than the FOV, then the signal aliasing, also known as wrap-around artifact, is encountered^44,45^. The part of the sample that lies beyond the edge of FOV is projected onto the other side of the image, which is a result of superposition of image signals from all the sample parts that are apart for different multiples of FOV in all imaging gradient directions. Signal aliasing can be prevented by oversampling the data, i.e., by scanning at the FOV larger than the sample size^46,47^. In most cases, signal aliasing is an unwanted effect as it causes image artifacts. This is the consequence of non-identical sample parts that are projected to the same image location. However, if the sample parts are identical, then the aliasing produces constructive superposition of the image signals. Theoretical analysis in 1D (presented in the next subsection) shows that such constructive superposition of aliased image signals is possible when the imaging is performed on a sample with translational symmetry, such that the imaging FOV ideally matches the unit cell of the sample. In that case, the resulting image signal is amplified by the factor equal to the number of identical objects in the unit cells of the sample. It is important to indicate that such signal amplification does not result in any increase of image noise. As shown in the schematic representation of the constructive signal aliasing (CSA) method in Fig. 1B: the MR imaging of a sample with 2D translational symmetry where the objects are arranged in a *M* × *M* matrix, results in the $M^{2}$-fold image signal increase without increase of image noise. The level of noise remains identical to that of the conventional MRI experiment on a sample consisting of only one object in the central unit cell if in both the experiments, the FOV is identical to the unit cell size and identical imaging method and hardware is used. Obviously, the increase in SNR of the CSA method over the conventional MRI without signal averaging is $M^{2}$-fold, which is still *M*-fold, when the signal averaging in the conventional MRI is equal to $M^{2}$ (Fig. 1A). The difference in scan times between the CSA method and the conventional method is then equal to $M^{2}$. An important difference between these two methods is also that the image obtained by the CSA method corresponds to an average object of the periodic sample, while the image obtained by the conventional MRI corresponds to an individual object.

### MRI theory of a periodic sample in 1 dimension

For a sample consisting of *M* periodically repeated identical objects $S_{1}$, the spatial distribution of the signal can be mathematically expressed by a convolution of the signal from the central object $S_{1} (x)$ with a set of *M* periodic Dirac delta functions represented by a function $P(x)$

1 $$ S_{M} (x) = S_{1} (x) * P(x). $$

here $S_{1} (x)$ is defined on a space $\left[ { - a/2,a/2} \right]$ and is zero elsewhere, while in $P(x)$ the delta functions are spaced by distance *a*, such that *M* is a positive odd number

2 $$ P(x) = \sum\limits_{m = - (M - 1)/2}^{(M - 1)/2} {\delta (x - ma)} ,\quad m \in {\mathbb{Z}}. $$

In the next step signal, $S_{M} (x)$ is transformed from *x*-space to *k*-space by continuous Fourier transformation. In this step, the convolution between the functions $S_{1} (x)$ and $P(x)$ in Eq. (1) transforms into a product of the corresponding Fourier transforms

3 $$ \hat{S}_{M} (k) = \hat{S}_{1} (k) \cdot \hat{P}(k). $$

which are equal to

4 $$ \hat{S}_{1} (k) = \int\limits_{ - a/2}^{a/2} {e^{ - ikx} S_{1} (x)\,dx} $$

and

5 $$ \begin{aligned} \hat{P}(k) & = \int\limits_{ - \infty }^{\infty } {e^{ - ikx} P(x)\,dx = } \int\limits_{ - \infty }^{\infty } {e^{ - ikx} \left[ {\sum\limits_{m = - (M - 1)/2}^{(M - 1)/2} {\delta (x - ma)} } \right]dx = } \sum\limits_{m = - (M - 1)/2}^{(M - 1)/2} {\left[ {\int\limits_{ - \infty }^{\infty } {e^{ - ikx} \delta (x - ma)\,dx} } \right]} = \\ & = \sum\limits_{m = - (M - 1)/2}^{(M - 1)/2} {e^{ - ikma} } = \frac{{\sin (k{\kern 1pt} aM/2)}}{{\sin (k{\kern 1pt} a/2)}}. \\ \end{aligned} $$

here symbol ^ denotes the Fourier transformed functions. In the MR imaging, the signals are not continuous functions, but discrete. For the signal sampled with *N* discrete points in the *k*-space separated by $\Delta k = 2\pi /FOV$; here the *FOV* is the size of the FOV. The discrete signal $\hat{S}_{M,j}$ is proportional to the Fourier transformed sample signal in the corresponding *k*-space points $\hat{S}_{M} (k_{j} )$

6 $$ \hat{S}_{M,j} = \frac{N}{FOV}\hat{S}_{M} (k_{j} ) = \frac{N}{FOV}\hat{S}_{1} (k_{j} ) \cdot \hat{P}(k_{j} ), $$

where $k_{j} = j\Delta k = 2\pi j/FOV$ and $j \, = - N/2 \ldots N/2 - 1$. The scaling constant of $N/FOV$ between discrete and continuous sample signals in *k*-space ensures the correct scaling and units of both functions. The image of the sample $S_{M,n}$ is therefore obtained for discrete points $x_{n} = n\,\Delta x = n\,FOV/N$, $n = - N/2 \ldots N/2 - 1$, by using inverse discrete Fourier transformation (DFT) of the signal $\hat{S}_{M,j}$ given in Eq. (6).

7 $$ S_{M,n} = \frac{1}{N}\sum\limits_{j = - N/2}^{N/2 - 1} {e^{i2\pi jn/N} \hat{S}_{M,j} } = \frac{1}{FOV}\sum\limits_{j = - N/2}^{N/2 - 1} {e^{i2\pi jn/N} \hat{S}_{1} (k_{j} ) \cdot \frac{{\sin (\pi j{\kern 1pt} aM/FOV)}}{{\sin (\pi j{\kern 1pt} a/FOV)}}} . $$

The last factor in Eq. (7) is akin the sinc function. The function has sharp peaks at the integer values of $j{\kern 1pt} a/{\text{FOV}}$ and comparatively much lower values elsewhere. For a general ratio $a/{\text{FOV}}$, the peak of the sine-like function is reached when *j* = 0, and possibly also with other values of *j* depending on the ratio. Consequently, in the DFT sum given by Eq. (7), the coefficients $\hat{S}_{1} (k_{j} )$ are heavily filtered by the sinc-like function favoring only the selected coefficients, within which $\hat{S}_{1} (0)$ dominates. Because of the filtering, the resulting reconstructed image is blurred and can be in the worst case represented only by the coefficient $\hat{S}_{1} (0)$ that corresponds to uniform image background. However, when *FOV* = *a,* the sinc-like function is equal to *M* for every *j*, so that the reconstructed image becomes equal to:

8 $$ S_{M,n} = \frac{M}{FOV}\sum\limits_{j = - N/2}^{N/2 - 1} {e^{i2\pi jn/N} \hat{S}_{1} (k_{j} )} = \frac{M}{N}\sum\limits_{j = - N/2}^{N/2 - 1} {e^{i2\pi jn/N} \hat{S}_{1,j} = M\,S_{1,n} } . $$

In this derivation the relation $\hat{S}_{1,j} = (N/FOV{)}\,\hat{S}_{1} (k_{j} )$ was used along with the inverse DFT definition. Resulting Eq. (8) is a theoretical confirmation that the signal undersampling with the FOV equal to the unit cell of the periodic sample results in signal amplification of the object image by factor equal to the number of objects in that sample.

## Results

Signal amplification in the MRM with the CSA method and its SNR properties were tested on a special test sample with 2D translational symmetry. The test sample consisted of a periodic pattern, i.e., JSI institutional logo arranged in a 3 × 3 matrix. The test sample was also used for studying the susceptibility of the CSA method for imaging the artifacts. Specifically, the effects of mismatch were studied between the imaging FOV and the unit cell size, as well as of the misalignment between the imaging axes and the symmetry axes on the imaging artifacts. Lastly, feasibilities of the CSA method were demonstrated on a non-phantom sample made of tablets arranged in periodic structure. This sample was scanned in 2D and 3D, and the obtained images were used to extract the moisture content distribution in an average tablet.

### Results on a test sample

**Figure 2:**
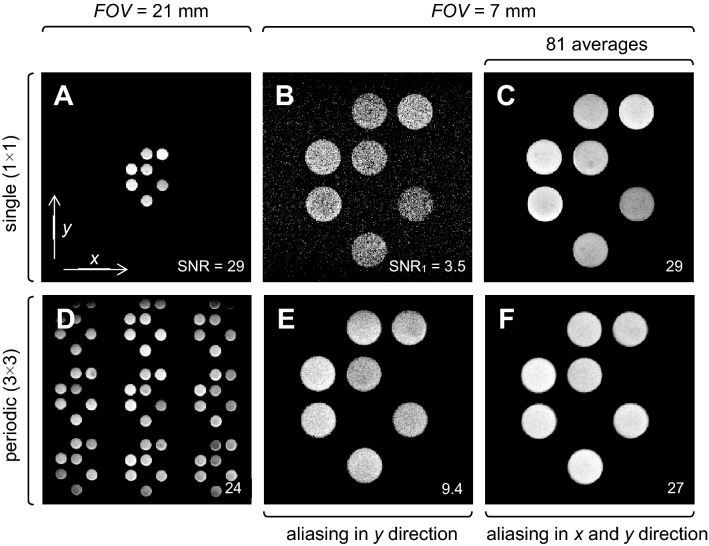
MR images of the test sample. (**A-C**) Images when only the central object was oil-filled and (**D-F**) images when all objects were oil-filled. (**A**, **D**) When the sample was scanned at *FOV* of 21 mm, SNR was 9 times higher than (**B**) that scanned at *FOV* of 7 mm (the voxel is reduced by a factor 9), and there was no signal amplification due to aliasing. (**E**) With signal aliasing only in the phase-encoding direction, the SNR increased by a factor of 3 and (**F**) when signal aliasing was present along both symmetry axes, SNR increased by a factor of 9 so that it reached the level of SNR before the *FOV* reduction. (**C**) Image resulting from 81 averages with standard scanning of the object at small *FOV*. The resulting image features about the same SNR as (**F**) the constructive aliasing approach with small *FOV* and no averaging.

Figure 2 depicts the results of MR imaging on the test sample with the JSI logo as an imaging object arranged in a 3 × 3 matrix. All experiments on the images (A-E) were done by the standard spin-echo (2DSE) imaging pulse sequence (Supplementary Fig. S3A), only the experiment shown on image (F) was done by the single point imaging (2DSE-SPI) pulse sequence (Supplementary Fig. S3B). In the experiments shown in the first row (A-C) only the central object was filled with oil so that the MR signal originated only from that object. In the experiment (A), the sample was scanned with the *FOV* of 21 mm (three times the unit cell size of 7 mm) so that the voxel size was equal to 82 µm and the obtained SNR image was equal to 29. In the experiment (B), the *FOV* was reduced to 7 mm (unit cell size) so that the voxel size decreased to 27 µm, and consequently the SNR decreased to 3.5, which is close to the theoretically expected ninefold SNR decrease (from 29 to 3.2). In order to regain the initial SNR value of 29, the last experiment had to be averaged 81 times, which was confirmed in the experiment (C).

In the experiments shown in the second row (D-F), all nine objects of the sample were filled with oil. In the experiment (D), where the *FOV* of 21 mm matched the sample size, the periodic structure of the sample can be clearly seen. In the experiment (E), the *FOV* was reduced to a single unit cell size (*a* = 7 mm) and the SNR decreased to 9.4. This result is a consequence of two competing effects, ninefold voxel size reduction and the threefold signal amplification due to the CSA effect in the phase-encoding direction. Therefore, the theoretically expected net effect is a threefold SNR decrease (from 29 to 9.7). In this experiment, the signal is aliased only in the phase-encoding direction because of the use of the SE imaging pulse sequence with antialiasing filter in the frequency-encoding direction. When this pulse sequence was replaced by the SPI pulse sequence in the experiment (F), the signal is aliased in both gradient directions so that the expected signal amplification due to CSA is ninefold, which is equal to the decrease in voxel size. Therefore, in theory, for this experiment (F) there should be no change in the SNR compared to the experiment (A). The corresponding experimental values of SNR of 27 in the experiment (F) vs. 29 in the experiment (A) confirm this prediction.

### Susceptibility to artifacts

**Figure 3:**
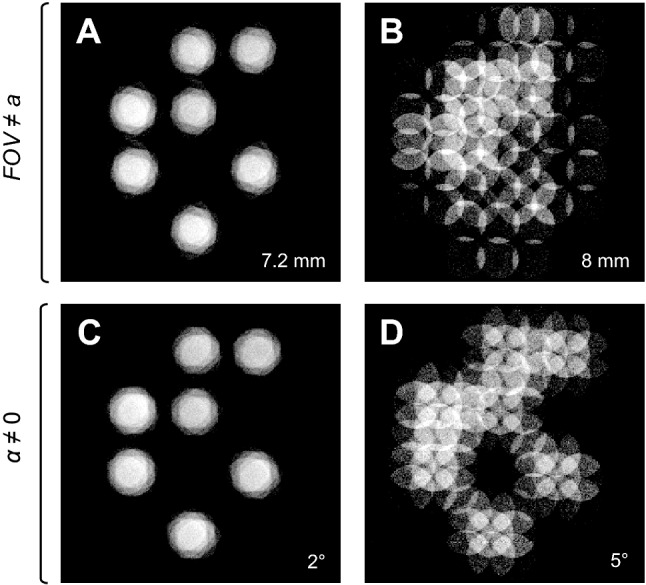
Sensitivity to imperfections in FOV and α. (**A**, **B**) Mismatch between the *FOV* and the unit cell size of the sample *a* and (**C**, **D**) misalignment between magnetic field gradient directions and symmetry axes of the sample measured by angle *α*. (**A**, **C**) The resulting images have small blurring when the discrepancies are small and (**B**, **D**) much higher blurring when *FOV* (**B**) and angle (**D**) are misadjusted to a more severe deviation.

The signal amplification with CSA is quite sensitive to experimental imperfections. Results of these are shown in Fig. 3 by examples of the CSA image in Fig. 2F in case of mismatch between the *FOV* and the unit cell size of *a* = 7 mm (A, B) and in case of discrepancy between directions of the imaging gradients and the sample’s symmetry axes for an angle *α* (C, D). When the imperfections are small (*FOV* = 7.2 mm or *α* = 2°), the shape of the object is still recognizable, however it is blurred. With higher discrepancies in angle and *FOV* (*FOV* = 8 mm or *α* = 5°), the object in the image is hardly recognizable and the CSA method fails. According to the theory (Supplementary Eqs. S1–S4), the mismatch between the field of view, the unit cell in size ($FOV \ne a$) and in orientation ($\alpha \ne 0$ ) results in the misalignment of objects in the CSA image that can be estimated by equation:

9 $$ \delta = (M - 1)\,a\sqrt {(1 - FOV/a)^{2} + \alpha^{2} } . $$

Misalignment *δ* is defined as the size of the region which maps the signal of the periodic sample with only a single point in the center of the unit cell. The misalignment can be considered also as the geometrical limit for the CSA image resolution. When there are no imperfections, the CSA image represents a spatial average of perfectly aligned objects (i.e. *δ* = 0). With the mismatch in *FOV* of 3% in the experiment (A), the misalignment increased to 400 µm, and it further increased to 2000 µm when the mismatch was 14% in the experiment (B). Similarly, with the mismatch in *α* for 2° in the experiment (C), the misalignment increased to 490 µm, and subsequently increased to 1220 µm when the mismatch was 5° in the experiment (D).

### MRM of a periodic sample of tablets

**Figure 4:**
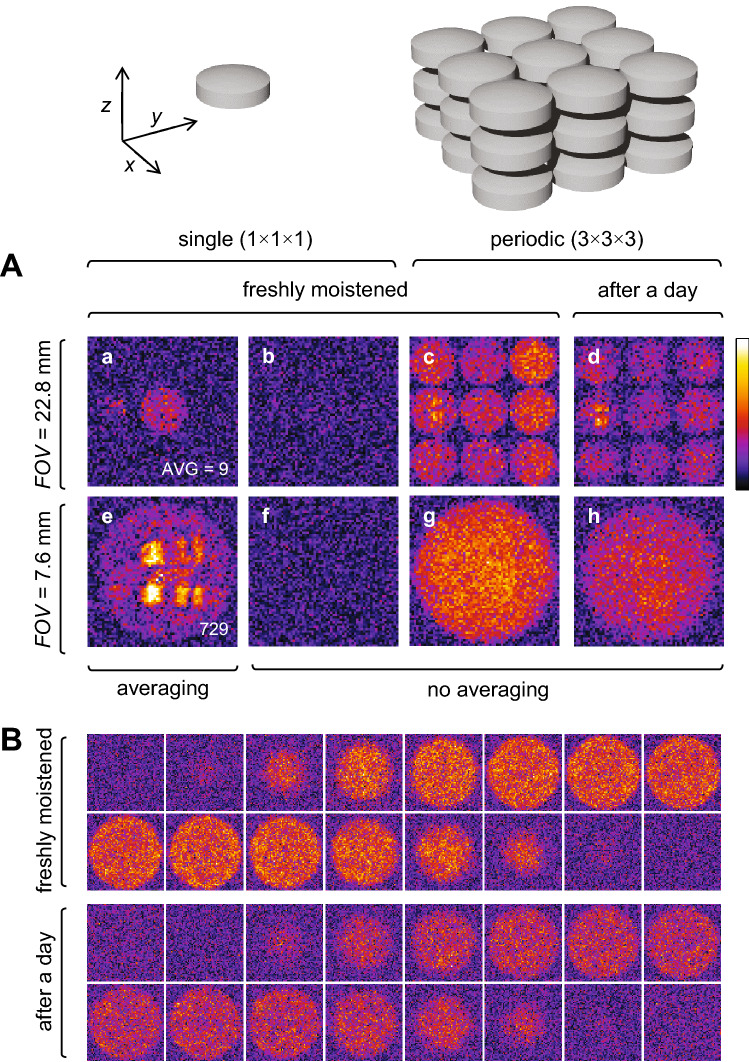
SPI images of tablets. (**A**) 2D SPI images of (a,b,e,f) a single tablet and of (c,d,g,h) a periodic sample of tablets at the *FOV* equal to: (c–d) triple and (e–h) single unit cell size. (b,f) The single tablet sample does not produce a detectable signal. (a,e) With sufficient averaging the signal becomes comparable to that of (c,g) the periodic sample without averaging. (d,h) After a day, this signal is lower due to moisture loss. (**B**) 3D SPI images of the periodic tablet sample freshly moistened and after a day.

Results of 2D SPI imaging on a sample of periodic tablets is shown in Fig. 4A. Firstly, standard approach on a single tablet sample without signal resulted only in noise with both *FOV*s, i.e., with the one equal to three times the unit cell size (*FOV* = 22.8 mm, experiment b) and to the unit cell size (*FOV* = 7.6 mm, experiment f). This is because the signal from the tablet was lower than the level of noise. However, when the periodic sample of 27 tablets was imaged with SPI using identical parameters, the SNR increased to 3 at *FOV* = 22.8 mm due to the signal co-addition along the *z*-direction and to SNR 27 at *FOV* = 7.6 mm due to the additional constructive signal aliasing in *x*- and *y*-directions. In the corresponding images at *FOV* = 22.8 mm (c) and *FOV* = 7.6 mm (g), array of tablets and also a single tablet are clearly visible due to the increase of the SNR. In theory, the images with the same SNR could be obtained also from a single tablet by signal averaging, i.e., 9 averages at *FOV* = 22.8 mm and 729 averages at *FOV* = 7.6 mm. While such image at *FOV* = 22.8 mm (a) has the expected SNR, this was not the case for the image at *FOV* = 7.6 mm (e). The scan time for this image was almost two days (42 h) and the tablet dried significantly during scanning so that the obtained SNR was much lower than in the corresponding experiment on the periodic sample, where the scan time was only 3.5 min. Images of the periodic sample obtained after a day (d, h), clearly indicate that sample lost significant amount of water and that drying was uniform.

The moisture distribution in an average tablet can be observed better in 3D SPI images of the periodic sample, in Fig. 4B. Again, it can be seen that the freshly moistened tablet sample (first row) contained considerably more moisture than the after a day (second row). To further improve SNR, the number of slices in Fig. 4B is reduced from 32 to 16 by averaging two adjacent slices; original 3D image stack in 32 slices is presented in (Supplementary Fig. S5). All SPI images of tablets, in 2D and in 3D, were contaminated by the signal of the RF probe itself. This problem was greatly reduced by the procedure described in (Supplementary Figs. S5, S6) so that in Fig. 4 are shown only the processed (decontaminated) SPI images.

**Figure 5:**
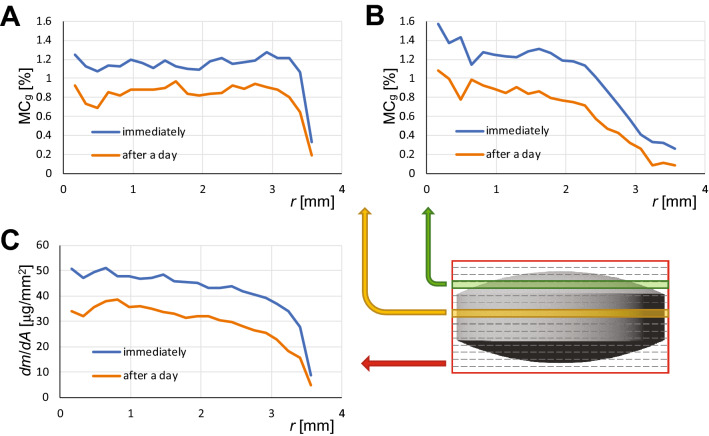
Radial profiles of moisture distribution in an average tablet. (**A**) and (**B**) radial profiles of gravimetric moisture content for central and off-center slice, respectively. (**C**) radial profile of water area density of the average tablet. Moisture distribution profiles correspond to tablets immediately after moistening (in blue) or after a day (in orange).

In Fig. 5A,B the radial profiles of gravimetric moisture content that correspond to the central slice (A) and to the off-center slice (B) of the tablet immediately and after a day after moistening are shown. These profiles were obtained by the analysis of the corresponding 3D SPI images. By similar analysis of 2D SPI images, the radial distribution of water area density in the tablet immediately and a day after moistening was obtained as well (shown in Fig. 5C). Unlike the moisture content, the water area density is also a function of the tablet’s shape.

## Discussion

In this study, a new method for the signal amplification in MRM is presented. This method utilizes the signal aliasing in MRI, also known as the wrap-around artifact. For a sample possessing the translational symmetry, the aliased signals are superimposed constructively when the sample is imaged with the FOV that ideally matches the sample’s unit cell both in size and orientation. Under these conditions, the SNR of the corresponding image is increased by the number of periodically repeating identical objects in the sample in comparison to the same experiment performed on the sample containing just a single object. This theoretical prediction was confirmed by experiments performed on the JSI logo test sample with 2D translational symmetry as well as on the 3D periodic sample of tablets. It was also demonstrated that the same SNR can also be obtained in a traditional experiment on the single-object sample with signal averaging, i.e., without the constructive superpositioning of the aliased signals. However, the number of signal averages in the experiment can soon become excessively large, as it must be equal to the squared number of objects in the sample. In the JSI logo experiment, where the sample consisted of 9 objects, this was already equal to 81; while in the tablet experiment, where the sample consisted of 27 objects, this number increased to 729.

By definition in the CSA method, theory suggests the SNR can be maintained constant while reducing the size of image voxels and proportionally reducing the size of objects, if the sample volume is maintained constant so that the sample contains increasingly smaller objects. In addition, the *B*_1_ coverage and the sensitivity of the RF probe must be homogeneous over the entire sample volume. However, it is very hard to maintain the ideal experimental conditions with smaller voxels using this approach. As shown by experiments in Fig. 3, where the image artifacts due to the experimental imperfections were demonstrated, the CSA method is very sensitive to the deviations in *FOV* and *α*. It is also important to consider that susceptibility to image artifacts increases proportionally with $M - 1$ factor, i.e., with the number of objects along a gradient direction minus one. Therefore, it becomes progressively harder to maintain optimal experimental conditions with an increasing *M* value. The origin of susceptibility to the artifacts can also be understood by the *k*-space signal analysis of the periodic samples, such that with an increasing number of unit cells in the sample, the signal becomes concentrated only at specific discrete points in the *k*-space that are apart by $2\pi /a$ (Eq. 5, Supplementary Fig. S1)^3^. Therefore, if the signal is not sampled in these points, the image has a reduced signal intensity and artifacts become apparent. The imperfections in the symmetry, e.g. poor periodicity due to the differences and unequal spacings among the objects also contribute to the possible artifacts, reducing the signal amplification as well. Another problem of the CSA method is associated with the gradient hardware design. If the magnetic field of gradient coils is not perfectly linear or the gradients along different directions are not perfectly orthogonal, the CSA method will not produce the expected results even if the sample has perfect translational symmetry and the settings of *FOV* and *α* are optimal. Usually the field linearity issues are bigger with larger *FOV*s. However, in the present experiments, no obvious issues associated with the field linearity were observed. The estimated field linearity was better than ± 1% peak to peak on a 30 mm sphere. The RF probe also did not produce homogeneous *B*_1_ field and therefore, its sensitivity was not homogeneous either. However, this RF probe imperfection did not result in geometrical distortions of the CSA image, it only caused slight imbalance among weights of the signals from different objects in the periodic sample. Similar effect can also be expected at higher fields due to the RF penetration problems.

In some cases, the object averaging property of the CSA method is highly desirable. One such example is already presented in this study by measuring the average moisture distribution in identical objects (tablets). With the standard approach one would need to image each object individually, then measure its moisture profile and in the end average all these profiles to get a statistically significant result. However, with the CSA approach, averaging is an inherent part of the method so that the moisture profile obtained CSA already corresponds to an average moisture profile of the studied objects. More importantly, the average moisture profile is obtained in a single scan instead of several scans (one for each object) and its corresponding SNR is much better (exactly by the square root of the number of objects) than the SNR of the profile obtained by averaging moisture profiles of the individual objects.

The CSA method performs well with phase-encoding while its implementation is more problematic with the frequency-encoding. One of the reasons for that is the use of frequency filter in signal acquisition such that its width is usually set to match the signal acquisition bandwidth. With such a setting, the signal aliasing is prevented in the frequency-encoding direction, thus the CSA method fails in this direction. Signal aliasing can also be made possible in the frequency-encoding direction by increasing the width of the frequency filter such that it passes through all frequencies of the sample. However, this option is problematic for CSA as it is difficult to ensure equal phase in all objects of the periodic sample. This phase uniformity is highly susceptible to any misalignment between centers of the *k*-space and of the signal acquisition window. All these aforementioned problems do not exist with the phase-encoding. Therefore, the pure phase-encoding methods (with phase-encoding in all spatial directions, e.g. SPI) are ideal for the CSA technique.

## Conclusion

The idea of using the wrap-around (aliasing) effect in the standard MRI was introduced in order to achieve the signal amplification for the imaging of samples with translational symmetry. The applicability of the concept was verified by simple experiments on test samples. It was demonstrated that the SNR remains unchanged when reducing the FOV to the unit cell if the total volume of all the unit cells from which the MR-signal is obtained is kept constant. This method is different from the traditional approach, where the SNR decreased proportionally to the voxel volume size. The missing loss of SNR with nominal smaller voxel volumes is a consequence of the summing of signal intensities from the different unit cells originating by aliasing. Thus, the obtained image represents a kind of spatially averaged image originating from several objects covered by the infolding of signal with difference to standard imaging at the same FOV, where there is information available from one single unit cell at reduced SNR. The practical applications support: a) artificially arranged sets of periodic samples, where the interest is not confined for the structure of the individual object but an average-type image of all objects (e.g. tablet imaging in Figs. 4,5), and b) the crystal-type structures mainly present in the solid-state bodies.

## Methods

### Samples

**Figure 6:**
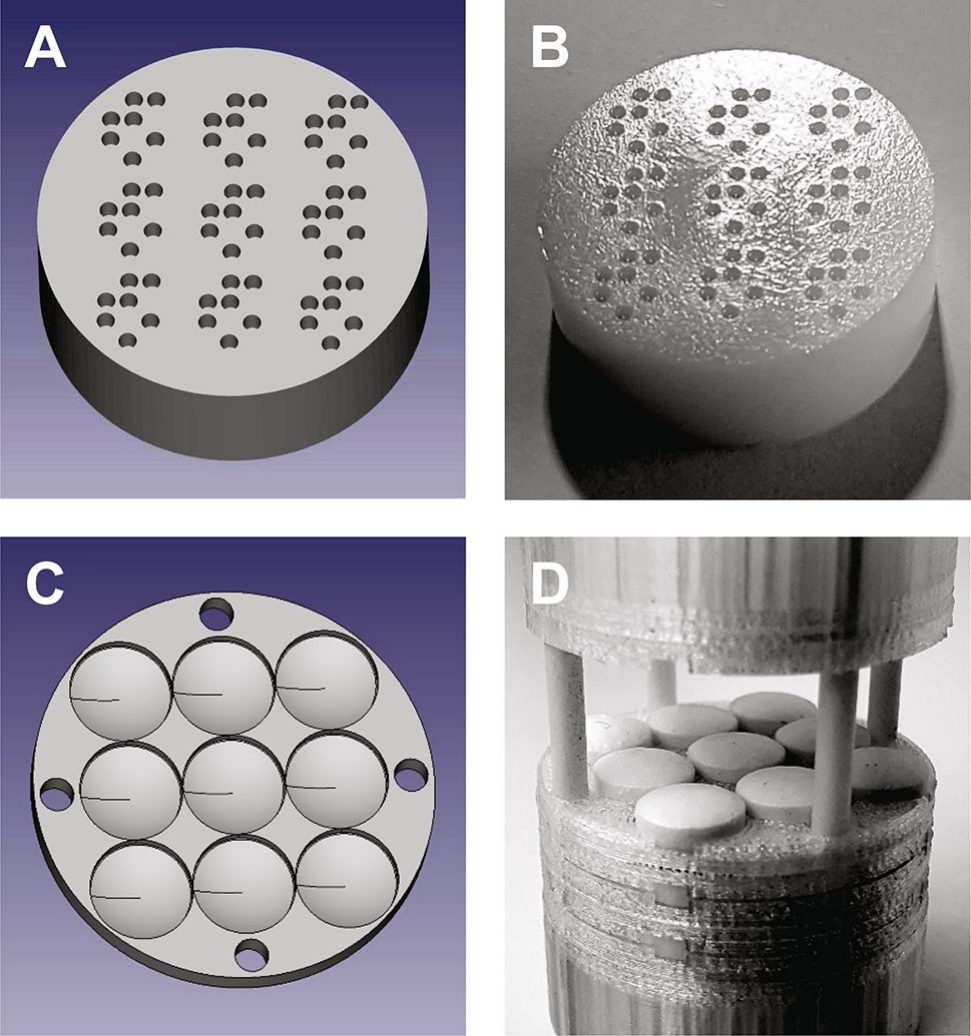
Tested periodic samples. (**A**) Computer model of the JSI logo test sample and (**B**) its realization made of POM plastic. 1.2 mm holes in the sample are oil-filled. The JSI logo sample has a 2D translational symmetry and consists of 9 unit cells of 7 × 7 mm^2^ in size that are arranged in a 3 × 3 matrix. (**C**) Computer model of a tablet holder segment, (**D**) partially opened sample holder loaded with 27 Aspirin Protect tablets in 3 layers. Each layer was covered from top and bottom with one holder segment. The periodic tablet sample has a 3D translational symmetry and 7.6 × 7.6 × 3.93 mm^3^ unit cells arranged in a 3 × 3 × 3 matrix.

To check the feasibility of the proposed CSA method, a special test sample with 2D translational symmetry was designed. On the face of a 27 mm diameter and 10 mm thick disc made of polyoxymethylene (POM) plastic, a repeated pattern of Jožef Stefan Institute’s (JSI) logo arranged in the form of an array of 3 × 3 matrix with the size 21 × 21 mm^2^ was carved to the depth of 2 mm. A logo that represented the unit cell of the sample had dimensions 7 × 7 mm^2^ and was made by drilling seven 1.2 mm diameter and 2 mm deep holes in the disc. Due to high precision required, the sample was made using a computer numerical controlled (CNC) machine following a 3D model (Fig. 6A), which was designed using the FreeCAD program. The holes in the sample were then filled with sunflower oil (Fig. 6B) that had NMR relaxation times *T*_1_ = 560 ± 30 ms and *T*_2_ = 230 ± 10 ms (measured at 400 MHz). A special care was taken to prevent the presence of air bubbles in the holes.

Practical use of the CSA method was demonstrated on a periodic sample made of 100 mg Aspirin Protected (Bayer AG, Leverkusen, Germany) tablets. Since the tablets are taken directly from the package and had too low signal for imaging, they were first immersed in water for a period of one hour then gently wiped to remove excess surface water and placed in a specially prepared tablet holder with the capacity of 27 tablets (Fig. 6C,D). The holder was CAD-designed based on actual tablet dimensions (Fig. S3) and then 3D printed from ABS plastic. It ensured precise and firm placement of the tablets in the body centers of a rectangular grid with the size of 3 × 3 × 3 and the unit cell of 7.6 × 7.6 × 3.93 mm^3^. NMR relaxation times of the moistened tablets were *T*_2_ = 217 ± 10 μs and *T*_1_ = 185 ± 20 ms and did not change much after a day (Supplementary Table S1), while their gravimetric and volumetric moisture contents were 1.2 ± 0.2 and 1.8 ± 0.3, respectively (Supplementary Table S2).

### MR imaging

Experiments were performed on a MR microscopy system consisting of a 9.4 T (400 MHz proton frequency) vertical wide-bore magnet (Jastec, Tokyo, Japan), a Bruker Micro 2.5 gradient system (Bruker, Ettlingen, Germany) and Tecmag Redstone spectrometer (Tecmag, Houston, TX, USA). All experiments were performed on a 30 mm quadrature Bruker RF probe with a 60 μs 90° RF pulse, running in the quadrature mode. After inserting the samples in the magnet, it was ensured that their symmetry axes were aligned with magnetic field gradient directions. The samples were scanned with different pulse sequences depending on their *T*_2_ relaxation time. Due to a longer *T*_2_ relaxation time, the JSI logo test sample was scanned with the spin-echo (SE) sequences, 2DSE and 2DSE-SPI (Fig. S1A,B), while due to much shorter *T*_2_ relaxation time, the periodic tablet sample was scanned with the single point imaging (SPI) sequences, 2DSPI and 3DSPI (Fig. S1C,D). All 2D pulse sequences had no slice selection gradient and use hard RF pulses for signal excitation and refocusing. The samples were scanned either with the *FOV* equal to the sample size (JSI logo *FOV* = 21 mm, tablet sample *FOV* = 22.8 mm) or to the unit cell size (JSI logo *FOV* = 7 mm, tablet sample *FOV* = 7.6 mm in *x-* and *y*-direction, *FOV* = 3.93 mm in *z*-direction). Other parameters for the JSI logo test sample were: flip angle *θ* = 40°, echo time TE = 6 ms, repetition time TR = 500 ms, imaging matrix 256 × 256, the signal filter matched acquisition bandwidth of 100 kHz (for the SE sequence); and for the periodic tablet sample: gradient stabilization time *t*_s_ = 200 μs, signal encoding time *t*_*p*_ = 200 μs, repetition time TR = 50 ms, imaging matrix 64 × 64 in 2D and 64 × 64 × 32 in 3D, number of signal averages NSA = 1 in 2D and NSA = 2 in 3D, scan time 3 min and 29 s in 2D and 3 h and 40 min in 3D. The tablet sample (single and periodic) was scanned immediately after moistening and one day after with identical methods.

### Moisture content profiles

Circularly symmetric intensity distribution in images of an average tablet allows calculation of radial intensity profiles, which was done using Radial Profile plugin for ImageJ (NIH, Bethesda, MD, USA). As the profiles were calculated from magnitude images with substantial noise, image intensity was not directly proportional to the signal and therefore to the moisture content in the tablet, but was elevated due of noise. If the noise level is known, the image intensity can be corrected by compensating the noise contribution. This compensation was done on the radial profiles by the procedure described in Supplementary material (Eqs. S8–S10), so that the corrected radial profiles were proportional to the NMR signal. Corrected profiles of 3D image slices were converted to the radial profiles of gravimetric moisture contents (*MC*_*g*_) by multiplying them with the ratio between the tablet’s average *MC*_*g*_ (Supplementary Table S2) divided by the tablet’s average corrected voxel intensity. Similarly, the radial profiles of water area density $dm/dA$, i.e., the mass of water per unit area as a function of radial distance *r*, was calculated from the corrected radial profile $S(r)$ of the 2D tablet image multiplied by the ratio between the absorbed water mass *m*, divided by the signal of the tablet (sum of all corrected pixel intensities of the tablet): $dm/dA = S(r)\;m/\int_{0}^{R} {S(r)\,2\pi r\,dr}$. Here *R* is the radius of the tablet.

## Supplementary Information

Supplementary Information.
