# Supplementary material for: Magnetic resonance microscopy of samples with translational symmetry with FOVs smaller than sample size
Source: Sci Rep. 2021 Jan 12;11:541. doi: 10.1038/s41598-020-80652-z (PMC7804297; doi:10.1038/s41598-020-80652-z)
Supplement: Supplementary file 1 — Supplementary Information. [file 41598_2020_80652_MOESM1_ESM.docx]

**Supplementary Material**

**Magnetic Resonance Microscopy of Samples with Translational Symmetry with FOVs Smaller than Sample Size**

**Igor Serša**^*^

Department of Condensed Matter Physics, Jožef Stefan Institute, Jamova 39, 1000 Ljubljana, Slovenia

^*^Correspondence and requests for materials should be addressed to I.S. (email: [igor.sersa@ijs.si](mailto:igor.sersa@ijs.si))

**Notes on the CSA method**

The described constructive aliasing "method" using smaller FOVs than sample size:

1. is restricted to severe periodically sub-structured samples and not only applied to them.
2. is limited to accurate coincidence of sampled k-space with the amount and direction to the symmetry axis vectors;
3. still demands for high gradient strength or encoding time corresponding to the highest spatial frequencies present in the elementary cell (hardware, i.e. gradient limitation);
4. demands for translational symmetry in the other two spatial dimensions if the FOV is smaller than the sample size in these other dimensions, relevant e.g. for
   1. the used spin echo sequence with no slice selective excitation in *z*- (slice) direction (Fig. 2E) and
   2. in *z*- and *y*-direction for pure phase encoding as e.g. for the used SPI sequence (Figs. 2F, 4) or - alternatively - the excitation of magnetization is limited in these additional directions by limited *B*_1_-field excitation;
5. is advantageous in simple practical application only for phase encoding dimension
6. gives structural information on an average elementary object composed of signal from several single objects and not for a single object; a structural deviation in a single object out of several, covered by the FOV might be not resolved due to the "averaging" principle by infolding connected the k-space sampling.
7. is featuring no loss in SNR against the cost of loosing information of the individual single object due to the summing up signal intensities from several objects as a consequence of the infolding of signal intensity from the several objects.

**MR and *k*-space image of a periodic sample**

Magnetic resonance images and their *k*-space signals are related by Fourier transform (FT). Usually, FT of a MR image, i.e., the *k*-space image, has in the center at *k* = 0 a high intensity signal that rapidly decreases to values close to zero in the outer *k*-space regions. The central part of the *k*-space contains low-frequency components that mainly define the image contrast, while the outer *k*-space regions contain high-frequency components which hold information needed to retain the image resolution. When a sample with translational symmetry is imaged (Fig. S1), a periodically repeating unit cell introduces a defined pattern with the *k*-space signal. To better observe this effect, only the central part of the *k*-space is shown. When the sample consists of only a single unit, such that there is no periodicity in the sample, the corresponding


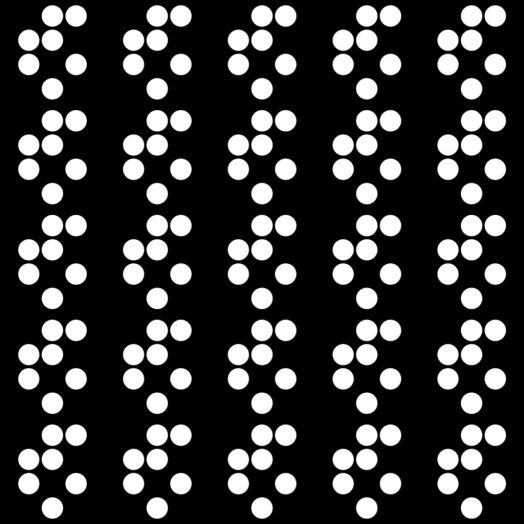

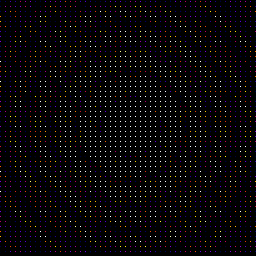

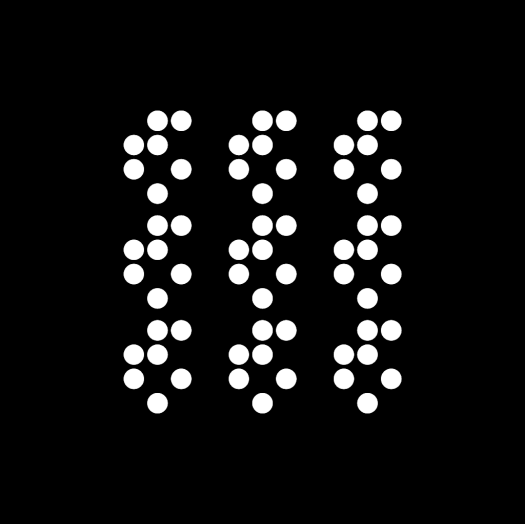

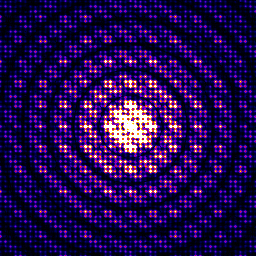

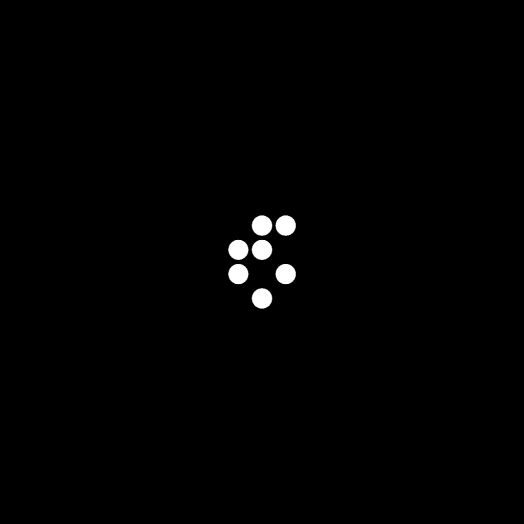

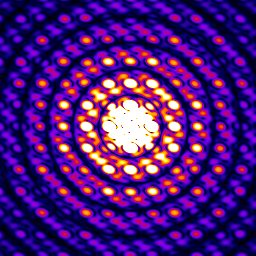


**Figure S1.** Effect of sample periodicity on the *k*-space image. Illustrated are the examples of: (1^st^ row) single-unit cell sample, (2^nd^ row) sample with unit cells arranged in a 3 by 3 matrix, (3^rd^ row) sample with unit cells arranged in a 5 by 5 matrix (left column images) and the corresponding *k*-space images (right column images).

*k*-space signal (Fig. S1, top row right) appears according to the explanation given above. However, when the unit cell repeats (Fig. S1, 2^nd^ and 3^rd^ row), the corresponding *k*-space signal looks as if an additional filter has been applied to the unit cell *k*-space signal. The filter functions as a multiplication of the signal with a grid-like function that passes through the signal at the junctions of the grid and suppresses all other signals. When the sample consists of more unit cells (Fig. S1, 3^rd^ row vs. 2^nd^ row) the grid constant does not change, only the filter gets sharper. Therefore, it appears that the grid constant is associated with the unit cell size and that a *k*-space signal of the sample consisting of an infinite number of periodically repeating unit cells is discrete, i.e., it is non-zero only in *k*-space points separated by the grid constant. This conclusion is verified also by mathematical analysis of a *k*-space signal of a periodic sample in 1D presented in the subsection “MRI theory of a periodic sample in 1 dimension” of the main manuscript.

**Misalignment of objects due to mismatch between the unit cell and FOV**

Figure S2A depicts a two-dimensional case when there is mismatch between the unit cell and the field of view both in the size and orientation. Field of view (FOV) is indicated by dashed lines, while the sample consists of small dots arranged in a square grid with the unit size of cell *a*. The grid is also tilted by angle $\alpha$ with respect to the field of view. For reference, there is also a third grid drawn in light gray with crossings in the middle of each field of view cell. The sample is positioned so that its green dot (the starting point) is in the center of the bottom-left cell. From the drawing can be inferred that the violet dot which is mostly to the right has already shifted from the cell center both in horizontal and vertical directions. The horizontal shift $\delta_{FOV}$ is mainly due to the mismatch between the field of view and the unit cell size ($FOV\neq a$), while the vertical shift $\delta_{\alpha}$ is due to the orientational mismatch ($\alpha\neq0$). The horizontal and vertical shifts can be mathematically expressed as

$\delta_{FOV}=(M-1)\left|a\cos(\alpha)-FOV\right|\approx(M-1)\left|a-FOV\right|$ , (S1)

$\delta_{\alpha}=(M-1) a\left|\sin(\alpha)\right|\approx(M-1) a\left|\alpha\right|$ . (S2)

Here, variable *M* denotes number of unit cells in one dimension. In addition, it was assumed that orientational mismatch is low ($\alpha\to0$) so that $\cos(\alpha)\approx1$ and $\sin(\alpha)\approx\alpha$; here angle $\alpha$ must be given in radians.

As can be seen from the simulated images in Fig. S4B,C, in both cases, i.e., the size mismatch as well as the orientation mismatch, image blurring is induced. The blurring appears in a form of a square region to which individual points are mapped. Size of this region, which can be also considered as the geometrical

*α*

FOV

FOV

*a*

*a*

*δ_FOV_*

*δ_α_*

**A**

**B**

*δ_FOV_*

*δ_FOV_*

**C**

**D**

*δ_α_*

*δ_α_*

*δ*

*δ_α_*

*δ_FOV_*

*δ_FOV_*

*δ_α_*

*β*

**Figure S2.** (**A**) Mismatch between the field of view and the unit cell in size ($FOV\neq a$) and in orientation ($\alpha\neq0$) resulted in misalignment of objects in the CSA image equal to (**B**) $\delta_{FOV}$ and (**C**) $\delta_{\alpha}$, respectively. (**D**) Misalignment of objects due to both the effects is equal to $\delta$.

resolution limit, is equal to $\delta_{FOV}$ in case of size mismatch (Fig. 4B) and is equal to $\delta_{\alpha}$ in case of orientation mismatch (Fig. 4C). The square region is in both cases aligned with the field of view with the green dot in the middle; however, its orientation is not identical. In the case of orientation mismatch, it is tilted by 90° with respect to the case of size mismatch. Figure S4D shows a general case when there is a mismatch between the field of view and the unit cell in size as well as the orientation. It this case, the image blurring also appears in a form of a tilted square region with size $\delta$.The tilt angle $\beta$ is determined by the ratio between $\delta_{\alpha}$ and $\delta_{FOV}$ as

$\beta=\arctan(\delta_{\alpha}/\delta_{FOV})$ , (S3)

while the misalignment of objects (geometrical resolution limit) is equal to

$\delta=\sqrt{\delta_{FOV}^{2}+\delta_{\alpha}^{2}}=(M-1) a\sqrt{(1-FOV/a)^{2}+\alpha^{2}}$ . (S4)

Equations S1-S4 are valid only for small deviations from ideal conditions (FOV ≈ *a* and *α* ≈ 0).

Image blurring given by Eqs. S1-S4 can be considered also as the geometric limiting factor to CSA image resolution. Other factors that can cause additional image blurring are image noise, sampling bandwidth, NMR relaxation, chemical shifts … In an ideal case, the resolution is limited by the voxel size, which is not included in the above equations for the resolution limit.

**Pulse sequences**

In the experiments four different imaging pulse sequences were used: two-dimensional standard spin-echo (2DSE, Fig. S3A), two-dimensional spin-echo version of the single point imaging (2DSE-SPI, Fig. S3B), standard two-dimensional single point imaging (2DSPI, Fig. S3C) and the standard three-dimensional single point imaging (3DSPI, Fig. S3D) sequence ^1, 2^. The SE sequences uses two different approaches for encoding the spatial information into the imaging signal, namely, the frequency and the phase encoding. With the frequency encoding approach, the signal is acquired in multiple time points in the presence of a constant magnetic field gradient switched on, so that signals originating from different points of the sample have different (position-dependent) frequencies. With the phase encoding, the signal is acquired only from a single point and without any magnetic field gradient switched on. However, prior to the signal acquisition, a magnetic field gradient of a certain amplitude is switched on for a constant time. In the absence of nuclear interactions, the two encoding approaches are equivalent if the magnetic field gradient time integral for the equivalent points of the two encoding approaches are the same. With nuclear interactions present, the two approaches are not equivalent as signals from different points of the frequency encoding approach experience different time evolution. In addition, the frequency encoding approach offers a possibility of adjusting the frequency bandwidth of signal acquisition. This is most often set to match the frequency bandwidth of discrete signal sampling so that signal aliasing is prevented ^3^. The phase encoding approach does not offer this control over the signal aliasing so that with this approach the signal aliasing is present whenever the sample size is larger than the FOV. The SPI sequences use the phase encoding in all spatial directions having as a consequence inherently present signal aliasing in all spatial directions. Another important advantage making SPI sequences ideal for the constructive signal aliasing method is that the signal is acquired at a constant time after the excitation. In case of the spin-echo SPI sequence (Fig. S3B), the acquired signal from all *k*-space points has no signal evolution and this is very low due to the short encoding times in the standard SPI sequences (Fig. S3C,D); therefore, the signals from different unit cells of the sample are identical and can therefore constructively superimpose ^2^.

**A**

**B**

π/2[x]

AQ[x]

*G_y_*

*G_x_*

*RF*

π[y]

echo

*TE*

**C**

π/2[x]

AQ[x]

*G_y_*

*G_x_*

*RF*

π[y]

echo

*TE*

*θ* [x]

AQ[x]

*G_y_*

*G_x_*

*RF*

encoding time

*t_p_*

*t_s_*

**D**

*θ* [x]

AQ[x]

*G_x_*

*RF*

*G_y_*

encoding time

*t_p_*

*t_s_*

*G_z_*

**Figure S3.** MRI pulse sequences: (**A**) two-dimensional spin-echo (2DSE), (**B**) two-dimensional spin-echo single point imaging (2DSE-SPI), (**C**) standard two-dimensional single point imaging (2DSPI) and (**D**) the standard three-dimensional single point imaging (3DSPI). All the two-dimensional imaging pulse sequences have no slice selection gradients and can be applied only to the samples with symmetry along the *z-*axis.

In the study 2DSE and 2DSE-SPI sequences were used to image the water-filled test sample with the JSI logo while 2DSPI and 3DSPI sequences were used to image the periodic sample made of tablets. In the SPI sequence the excitation pulse *θ*[x] was executed with a stabilization delay *t_s_* after phase encoding gradients were switched on. This was needed in order to have stable magnetic field gradients during the encoding period. In order to maximize signal per scan time, the flip angle *θ* was equal to the Ernst angle, i.e., $\theta=\arccos(\exp(-TR/T_{1}))$.

**MR imaging of tablets**

The method of constructive signal aliasing was demonstrated on a periodic sample of 100 mg Aspirin Protect tablets (Bayer AG, Leverkusen, Germany). The Aspirin tablet is round with spherical surfaces at the top and at the bottom. It also has no engravings so that it is perfectly cylindrically symmetric. From the tablet’s physical dimensions (Fig. S4), its volume of 89.3 mm^3^ was estimated.


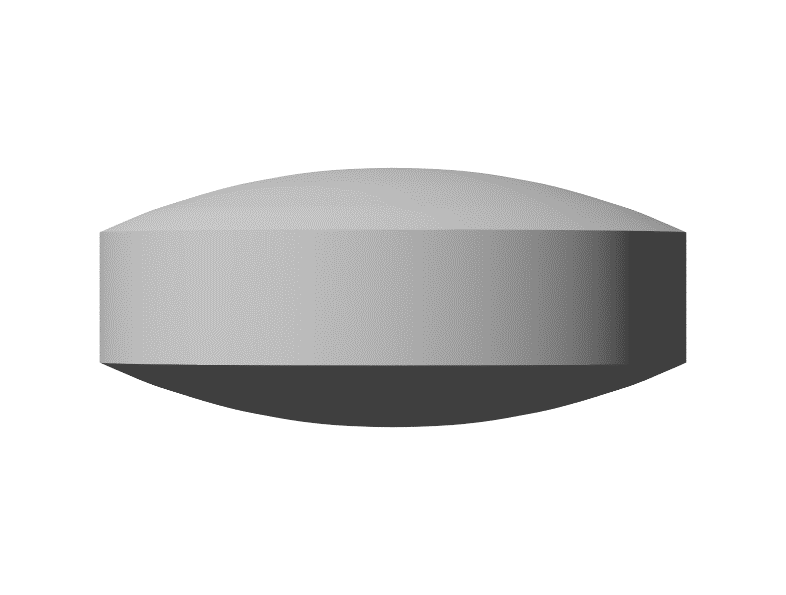


3.2 mm

7.3 mm

1.6 mm

**Figure S4.** Dimensions of the tablet.

Prior to imaging, the NMR relaxation times of the moistened tablets were measured (Table S1). Relaxation time *T*_1_ was determined from the best fits of the model function $S(TR)= S_{0}(1-\exp(-TR/T_{1}))$ to the amplitudes of the signal after excitation with a 90 ° RF pulse at different repetition times TR. Relaxation time *T*_2_ was determined from the spectral line width at half maximum (FWHM value) as $T_{2}=1/(\pi\text{FWHM})$ .

| NMR relaxation time | immediately | after a day |
| --- | --- | --- |
| *T*_2_ [μs] | 217±10 | 238±10 |
| *T*_1_ [ms] | 185±20 | 203±23 |

**Table S1.** NMR relaxation times of the tablets immediately after moistening and after a day.

In addition, the amount of absorbed water was determined by weighting the tablets before and after moistening (Table S2).

| tablet sample | weight [mg] | | | MC_g_[%] | MC_v_[%] |
| --- | --- | --- | --- | --- | --- |
|  | before moistening | after moistening | absorbed water |  |  |
| 1 | 136.4 | 137.7 | 1.3 | 0.95 | 1.46 |
| 2 | 136.2 | 138 | 1.8 | 1.32 | 2.02 |
| 3 | 139.1 | 140.8 | 1.7 | 1.22 | 1.90 |
| average | 137.2±1.6 | 138.8±1.7 | 1.6±0.3 | 1.2±0.2 | 1.8±0.3 |

**Table S2.** Tablet weight before and after moistening in water. These two weights along with the tablets volume enabled the calculation of the water absorbed, also of the gravimetric (MC_g_) and volumetric (MC_v_) moisture contents were estimated.

The approximate measured value of *T*_1_ = 200 ms was used to determine the optimal excitation angle, i.e., Ernst angle. This was equal to *θ* = 40° and corresponds to the excitation RF pulse of 25 μs. Gravimetric moisture content (MC_g_) was calculated as 100 times the difference between the tablet weight after (*m_wet_*) and before (*m_dry_*) moistening divided by the tablet weight before moistening:

$MC_{g}\left[\%\right]=100 \frac{m_{wet}-m_{dry}}{m_{dry}}$ . (S5)

While the volumetric moisture content (MC_v_) was calculated as 100 times the absorbed water volume (*V_water_*) divided by the wet tablet volume (*V_wet_*).

$MC_{v}\left[\%\right]=100 \frac{V_{water}}{V_{wet}}$. (S6)

The signal detected from the tablet sample was low. This was due to the NMR relaxation effects and also due to the low concentration of water in the tablets. The effect of the NMR relaxation on the signal reduction can be estimated using the formula:

$S_{SPI}=S_{0} \frac{1-\exp(-TR/T_{1})}{1-\cos(\theta)\exp(-TR/T_{1})} \exp(-t_{p}/T_{2}) \sin(\theta)$ , (S7)

Here the SPI sequence parameters were: *t_p_* = 200 μs, TR = 50 ms, *θ* = 40° and the NMR relaxation times are given in Table S1. For the sample immediately after moistening, this signal reduction is equal to $S_{SPI}/S_{0}=0.15$. The amount of water absorbed in the tablet was 1.6 mg which corresponds to the volumetric moisture content (MC_v_) of 1.8 % (Table S2). Both factors combined therefore resulted in


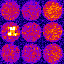

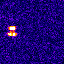

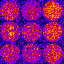

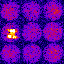

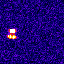

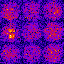

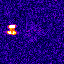

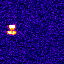

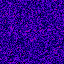

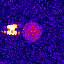

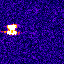

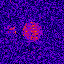


empty RF probe

RF probe with sample

subtracted
(1^st^-2^nd^ column)


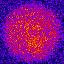

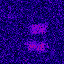

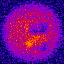

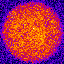

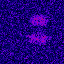

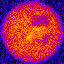

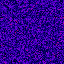

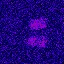

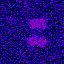


single tablet
fresh
FOV = 22.8 mm
AVG = 9

single tablet
fresh
FOV = 22.8 mm
no averaging

single tablet
fresh
FOV = 7.6 mm
no averaging


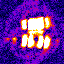

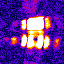

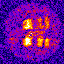


single tablet
fresh
FOV = 7.6 mm
AVG = 729

3×3×3 tablets
after a day
FOV = 22.8 mm
no averaging

3×3×3 tablets
fresh
FOV = 22.8 mm
no averaging

3×3×3 tablets
fresh
FOV = 7.6 mm
no averaging

3×3×3 tablets
after a day
FOV = 7.6 mm
no averaging

**Figure S5.** 2D SPI images of a single and periodic tablet sample.


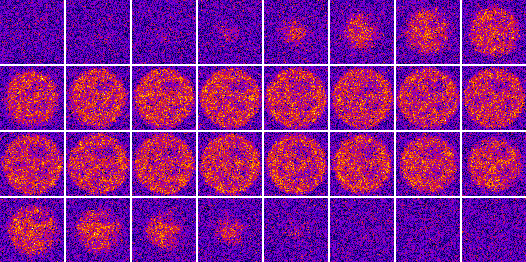

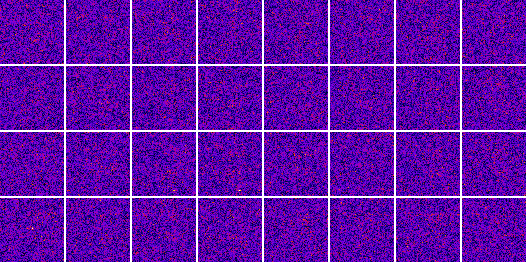

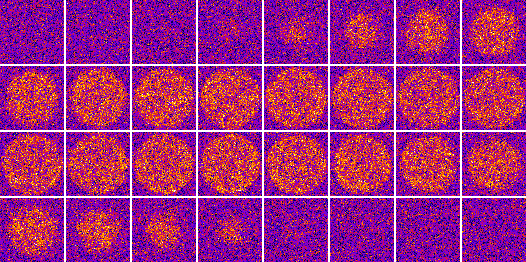

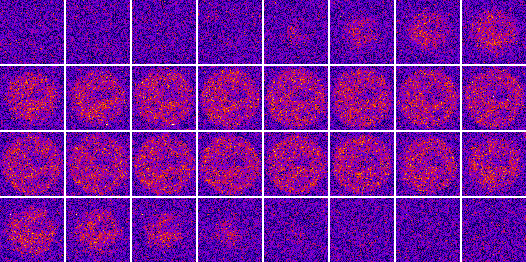

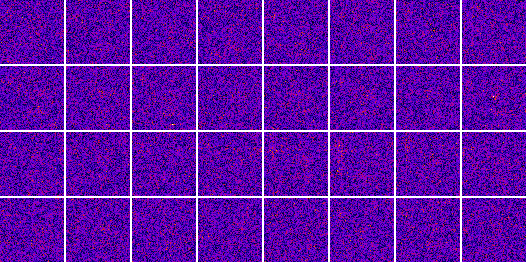

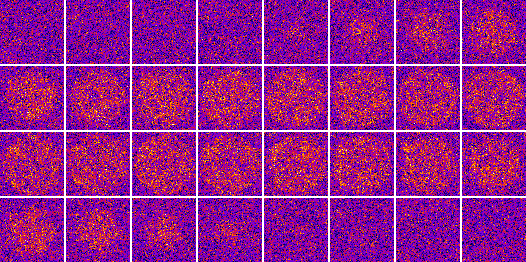


RF probe with sample

empty RF probe

subtracted images

freshly moistened sample

after a day

**Figure S6.** 3D SPI images of a single and periodic tablet sample.

approximately 370-fold signal reduction compared to the amount of signal that would be received from the bulk water sample at $t_{p}\ll T_{2}$ and $TR\gg T_{1}$ using the same method and hardware.

Due to the short *T*_2_ relaxation time of the tablets, SPI was performed with the short signal encoding time *t_p_* = 200 μs. However, with this encoding time, a considerable amount of the detected signal was coming from the RF probe itself, most likely from the glue and other semisolid materials used in the construction of the probe. This signal in SPI imaging was superimposed to the tablet signal and thus introduced image artifacts, which were more pronounced also because of the low tablet signal. The effect was especially significant when imaging a single tablet sample or when the tablets became dry such that the signal of the probe became higher than of the sample. In the study this effect was compensated by the following procedure. First, a SPI image of the sample was acquired. Then the sample was removed from the RF probe without removing the probe from the magnet or any probe repositioning or reorienting. Then a SPI image of the empty probe was acquired using the same SPI sequence and parameters. In the final step, the image of the empty probe was subtracted from the image of the sample. The subtraction was done in a phase sensitive mode so that complex signals were subtracted. This procedure is illustrated in Fig. S5 with 2D SPI images of the sample (left column), of the empty probe (middle column) and of the subtraction image (right column). In Fig. 4A of the main manuscript, only the final image results are shown that are identical to these in the right column. It can be seen that the original (unprocessed) images of the sample have considerable artefacts due to the NMR signal of the empty probe. Subsequently, this does not always increase the sample signal, but it may also decrease it due to the phase-sensitive nature of the signal. It can also be seen that the image subtraction procedure is quite efficient in suppressing the artifacts, however, at the price of an increased image noise. This is higher for the factor of $\sqrt{2}$ compared the source images. The same procedure was applied also to 3D SPI images to the periodic tablet sample in Fig. S6. Original images of the sample are less noisy than the corresponding subtracted images, however, they have artifacts due to the empty probe signal. This cannot be seen to that extent on the subtracted images.

**Image intensity correction for the background noise**

Magnetic resonance images are mostly magnitude images, meaning that amplitude of the phase-sensitive NMR signal is presented and phase information is omitted. This always has a consequence on positive image intensity *I*. In cases where no signal is detected ($S=0$), so only noise is present in the image, the average intensity is not zero but is equal to $\bar{I}=\sigma\sqrt{\pi/2}$, where $\sigma$ is standard deviation of the normally distributed noise in a phase-sensitive signal. The result is similar also when the signal is lower or comparable to the noise ($S\leq\sigma$) so that average intensity of the magnitude image is then not equal to the signal but becomes considerably higher ($\bar{I}>S$). Only when the signal is much higher than noise ($S\gg\sigma$) average intensity of the magnitude image becomes equal to the signal ($\bar{I}=S$). The probability distribution for the image intensity *I* as a function of signal *S* and noise $\sigma$ is given by Rice distribution ^4^

$P(I)=\frac{I}{\sigma^{2}}\exp\left(-\frac{I^{2}+S^{2}}{2\sigma^{2}}\right)I_{0}\left(\frac{IS}{\sigma^{2}}\right)$ , (S8)

where *I*_0_() is the modified Bessel function of the first kind with order zero. As already noted above, the distribution gives an elevated average intensity $\bar{I}$ in case of signal *S* with noise $\sigma$

$\bar{I}=\sigma\sqrt{\frac{\pi}{2}} L_{1/2}\left(-\frac{S^{2}}{2\sigma^{2}}\right)$ . (S9)

**Figure S7.** Radial profiles of the tablet image intensity before (dashed line) and after (continuous line) the correction with Eq. S10. Profiles in panels (A), (B), (C) correspond to equally labeled panels of Fig. 5.

*r* [mm]

*r* [mm]

*r* [mm]

Intensity [a.u.]

Intensity [a.u.]

Intensity [a.u.]

**A**

**B**

**C**

Here $L_{1/2}()$ is Laguerre polynomial. Equation S9 can be inverted so that the signal can be expressed as a function of noise and average intensity

$S=\sigma\sqrt{-2L_{1/2}^{-1}\left(\sqrt{\frac{2}{\pi}} \frac{\bar{I}}{\sigma}\right)}$ , (S10)

here $L_{1/2}^{-1}()$ is the inverse Laguerre polynomial.

Ratios between the image intensities and noise in SPI images of tablets from Fig. 4 are in the range between 2.1 (Fig. 4B, after a day) and 6 (Fig. 4Ag). According to Eq. S9, the intensities in these images are somewhat higher than the signal and are therefore not proportional to the moisture content. However, after applying the correction given by Eq. S10 to these intensities, the elevation of intensities due to noise is compensated so that they become proportional to the signal and also to the moisture content. In the study the intensity correction was not done on the images directly but to their measured radial profiles. Intensity correction of the radial profiles by Eq. S10 was implemented in Mathematica (Wolfram Research, Champaign, IL, USA). In Fig. S7 for the continuous lines, the same three intensity corrected radial profiles from Fig. 5 are shown. In addition to these profiles, the dashed lines represent their corresponding profiles before the intensity correction was applied. It can be seen that the difference between the original and corrected profiles is especially large when the signal is low, while the differences diminishes when the signal is high.

**Supplementary Material References**

1. Vlaardingerbroek MT, den Boer JA. *Magnetic resonance imaging : theory and practice*. Springer (1996).

2. Callaghan PT. *Principles of nuclear magnetic resonance microscopy*. Oxford University Press (1991).

3. Pusey E, Yoon C, Anselmo ML, Lufkin RB. Aliasing artifacts in MR imaging. *Comput Med Imaging Graph* **12**, 219-224 (1988).

4. Bonny JM, Renou JP, Zanca M. Optimal Measurement of Magnitude and Phase from MR Data. *J Magn Reson B* **113**, 136-144 (1996).
